# Education and Cohabitation in Britain: A Return to Traditional Patterns?

**DOI:** 10.1111/j.1728-4457.2013.00611.x

**Published:** 2013-09-11

**Authors:** Máire Ní Bhrolcháin, Éva Beaujouan

**Affiliations:** 1 Professor of Demography, Division of Social Statistics and Demography University of Southampton, UK.; 2 Research Scientist, Vienna Institute of Demography Austria.

## Abstract

Cohabitation is sometimes thought of as being inversely associated with education, but in Britain a more complex picture emerges. Educational group differences in cohabitation vary by age, time period, cohort, and indicator used. Well-educated women pioneered cohabitation in Britain in the 1970s and 1980s. In the most recent cohorts, however, the less educated have exceeded the best educated in the proportions ever having cohabited at young ages. But the main difference by education currently seems largely a matter of timing—that is, the less educated start cohabiting earlier than the best educated. In Britain, educational differentials in cohabitation appear to be reinstating longstanding social patterns in the level and timing of marriage. Taking partnerships as a whole, social differentials have been fairly stable. Following a period of innovation and diffusion, there is much continuity with the past.

The high and rising prevalence of cohabitation in many developed countries has put the phenomenon at the forefront of discussion and debate on family change. Contemporary cohabitation, which dates primarily from the 1960s and 1970s, has attracted much attention as a demographic and social innovation.^1^ Demographic interest hinges on cohabitation as an informal co-residential union that is less well defined and documented than marriage, its traditional counterpart. The role of cohabitation in the modern family is the focus of sociological attention—in particular, how closely it resembles marriage or such premarital statuses as dating and formal engagement (Smock 2000; Heuveline and Timberlake 2004). Family change is a politically contentious subject in both Britain and the United States. Cohabitation has entered the policy debate regarding legal provision for cohabiters and the suitability of cohabiting unions for the rearing of children.^2^ Sharp divisions occur, between and within political parties and among the public at large, on the acceptability of cohabitation as a living arrangement and on the justification for government policy favoring or promoting marriage.^3^

Beyond its relevance to contemporary policy debate, an understanding of how cohabitation varies among social groups is essential for an appreciation of its origins and contemporary significance. In this article we examine whether and how cohabitation has differed among educational groups in Britain since the 1970s. Educational differentials are of particular interest because education encapsulates several aspects of advantage, being closely linked with labor market prospects, earning potential, social status, and cultural outlook.

## Existing evidence

Previous findings on the relationship between cohabitation and education are not altogether consistent between sources (Carmichael 1995; Kravdal 1999). Several studies, especially recent ones from the US, have reported an inverse association between cohabitation and education.^4^ By contrast, other sources, both American and European, have found a higher frequency of cohabitation among the better educated.^5^ In addition, numerous multivariate analyses, relating to the UK, the US, and other developed societies, report a net association between education and cohabitation that is either positive or not significant.^6^ The covariates in these multivariate analyses differ from one study to the next; some include time-varying educational enrollment along with a measure of educational level. Several studies have found that in earlier decades cohabitation was more common among the better educated or among young people of higher-status backgrounds, but that over time educational and socioeconomic groups either converged or crossed over.^7^ Finally, some authors conclude that there is either no systematic relationship or only a weak one between education and cohabitation.^8^

The approaches adopted in previous studies have been diverse. Investigations vary in the methods and measures used, in the age groups examined, and in temporal coverage. The range of indicators employed includes: current cohabitation; ever having cohabited; proportion of first or of current unions that are a cohabitation; proportion of persons marrying who cohabit beforehand; and coefficient on education, net of a range of covariates, sometimes including enrollment, in models with various specifications of cohabitation as the dependent variable.^9^ Methods range from descriptive tables or graphs to regression analysis of various kinds. Some studies examine only young women, and others a single age group, whether narrowly or broadly defined.^10^ Finally, some investigations are based on a single cohort or cross-section, while others examine a range of cohorts or period cross-sections. Each of these methodological choices affects the direction and size of the measured association between education and cohabitation. This being so, it is not surprising that previous accounts of the link between education and cohabitation differ.

## Data and methods

Our data are from a combined file of annual rounds of the British General Household Survey (GHS) for the years 1979–2007. Near-complete histories of both marriage and cohabitation were collected only from GHS rounds 2000–01 onward, so our study is confined to those rounds (Beaujouan and Ní Bhrolcháin 2011).^11^ The partnership histories have been validated internally and against external sources (Berrington et al. 2011). The sample in each annual survey is of women aged 16–59 resident in private households.^12^ All analyses use a new set of weights constructed specifically for analyzing the Family Information section of the GHS from 1979 to 2007 (Beaujouan, Brown, and Ní Bhrolcháin 2011).

We classify educational level using the age at which respondents first completed continuous education. The indicator has the dual advantage of being strongly correlated, in the UK, with educational attainment while also remaining fixed throughout the life course. People who initially leave and subsequently return to education are classified by the age at which they originally left continuous education, thus removing a potential source of endogeneity (Kravdal 2004; Hoem and Kreyenfeld 2006). This approach is particularly important in relation to cohabitation at younger ages.^13^

Our methods are descriptive and graphical. We consider only the straightforward, gross relationship between education and cohabitation, rather than the net association adjusting for other factors. It is this relationship that is of primary interest in a policy context. For simplicity, we focus on the lowest and highest education groups—women who left continuous education at ages 13–17 and at ages 21+. Of those completing their education in 1980–84, 65 percent left at ages 13–17 and 14 percent at 21+. The corresponding figures in 2000–04 are 37 percent and 38 percent, reflecting the sizable expansion in educational participation over the period. The term “partnership” is used throughout to refer to cohabitation and marriage together—that is, to informal or formal co-residential unions.

## Findings

Table 1 indicates that the growth in the cumulative incidence of cohabitation has occurred at all educational levels.^14^ At the start of the period, the best-educated women aged 25+ had far higher proportions who had ever cohabited than the low education group.^15^ The middle education group tend to be intermediate. The cumulative frequency of cohabitation increased more rapidly in the low education group, and by 2000–04 they had overtaken the most highly educated in all but the oldest (35–39) age group shown. We return to this feature below.

**TABLE 1:** Percent of women who ever cohabited, by age and age at completing education: Great Britain, 1980–84 and 2000–04

	**Early leavers (13–17)**		**Middle leavers (18–20)**		**Late leavers (21+)**	
**Women’s age**	**1980–84**	**2000–04**	**1980–84**	**2000–04**	**1980–84**	**2000–04**
20–24	18.3	49.6	12.9	35.9	17.0	30.8
25–29	19.1	69.3	20.9	59.1	33.1	54.6
30–34	14.6	70.1	19.4	65.9	29.8	67.4
35–39	12.8	60.3	16.7	58.6	21.6	63.5

NOTE: For confidence intervals, see Ní Bhrolcháin and Beaujouan (2013). Sample comprises women answering the Family Information section of the GHS 2000–07 who had a valid partnership and fertility history.

SOURCE: CPC GHS time-series data file.

**FIGURE 1:**
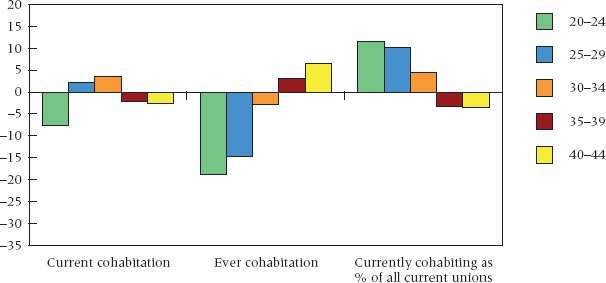
Educational differentials in cohabitation by age, using three measures: current cohabitation, ever cohabited, and cohabitation as a percentage of current unions, Great Britain 2000–04 NOTE: Plotted here are the differences between late leavers and early leavers; positive figures thus reflect a higher frequency among late leavers and negative figures a higher frequency among early leavers. For confidence intervals, see Ní Bhrolcháin and Beaujouan (2013). SOURCE: Same as Table 1.

Assessments of the link between education and cohabitation often take a cross-sectional approach (Chandra et al. 2005; Kennedy and Bumpass 2008; Esteve, Lesthaeghe, and López-Gay 2012), and so we begin with some data for the period 2000–04. To illustrate the educational differentials in cohabitation, we look at three indicators of cohabitation commonly used: the proportion currently cohabiting, the proportion ever having cohabited, and the proportion cohabiting among those currently in a union. Educational differentials (late school leavers minus early school leavers) in each of these measures are shown in Figure 1 by age. The figure has two prominent features. First, social differentials vary by age for all three indicators. Second, the size, and in some cases the direction, of group differences vary between indicators. On some indicators and in some age groups, an inverse relationship is found in these British data, but on other measures or in other age groups, the association is positive. All of these indicators have valid uses, but they measure different things. Differentials in current cohabitation reveal the state of group differences at a point in time and are the net outcome of moves into and out of cohabitation. One could argue that this picture of current status is of greatest relevance for policy purposes, as it is a snapshot of the position at any given time. On the other hand, unlike the proportion ever cohabitating, current differentials say little about past experience, and are therefore less informative from an explanatory perspective.

**FIGURE 2:**
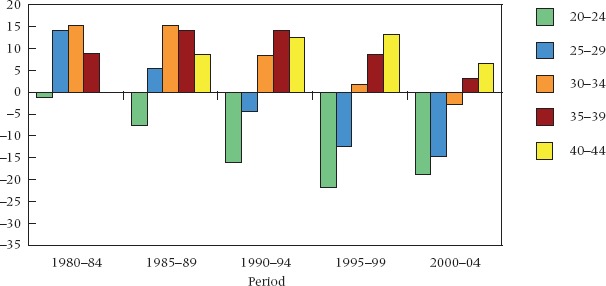
Educational differentials in cumulative incidence of cohabitation by period and age, Great Britain, 1980–84 to 2000–04 NOTE: See note to Figure 1. SOURCE: Same as Table 1.

Figure 2, which is confined to ever cohabited, plots educational differentials by age from 1980–84 to 2000–04. At the start of the period, substantially higher proportions of the best educated women aged 25+ had ever cohabited. In 1980–84, between 9 percent and 15 percent more of the best than of the least educated women aged 25+ had experienced cohabitation. By 2000–04, however, the differential has reversed at younger ages, and only in the 40–44 age group did the best educated women have a significantly higher cumulative frequency. At ages 25–39, that is, an initially positive gradient either vanishes or becomes increasingly negative. Over the same period the 20–24 age group initially shows no difference between education groups, but a sizable gap then opens up, reaching 19 percent by 2000–04. In sum, we see sizable differentials in the cumulative incidence of cohabitation by age and a substantial change in the age patterns across these two and a half decades.

**FIGURE 3:**
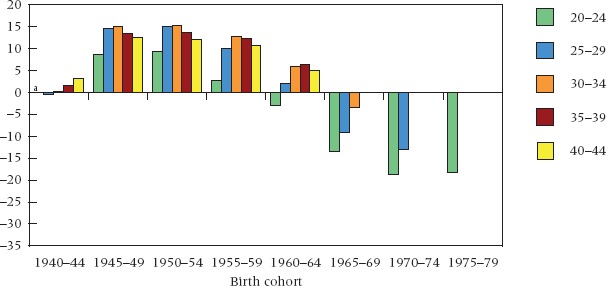
Educational differentials in cumulative incidence of cohabitation by birth cohort and age: Great Britain, cohorts 1940–44 to 1975–79 NOTE: See note to Figure 1. ^a^Data unavailable for ages 20–24 in 1940–44 birth cohort. SOURCE: Same as Table 1.

The dynamic underlying the shifts over time in Figure 2 becomes clearer when these are viewed in cohort mode, as is done in Figure 3. Two features stand out, one in cross-cohort comparison and the other within cohorts. Across cohorts, the differential in cumulative experience of cohabitation shifts dramatically, with a higher frequency among the best educated in the early cohorts up to the mid-1960s, and among the least educated in the cohorts of 1965–69 and after. On this evidence, educated women led the trend to nonmarital cohabitation in Britain. They accumulated a greater frequency of cohabitation than the less educated at young ages in the pre-1960s cohorts and maintained a relatively fixed lead up to ages 40–44. This turned around in the more recent cohorts, with levels of cohabitation among early school leavers progressively approaching those among the late leavers and overtaking them at younger ages. The data suggest a widening of the gap at younger ages in recent cohorts, especially at ages 20–24. Such large differences at young ages are what one would expect in relation to any type of partnership: better-educated women are either enrolled in education at these ages or have only recently completed their schooling, hence they are less advanced in their partnership experience than the low education group who leave education early.

Within cohorts, we see a second transformation. In the older generations, the higher incidence of cohabitation among the best educated remains relatively fixed with rising age. By contrast, in more recent cohorts, the education gap in cumulative incidence diminishes with rising age (though confidence intervals overlap in some cases: see Ní Bhrolcháin and Beaujouan 2013: Figure 5). The signs, therefore, suggest that education group differences in cumulative cohabitation in recent cohorts will ultimately reflect largely a timing effect: that less-educated women now cohabit in greater proportions at younger ages, but that the better educated have caught up on reaching their early 40s, having entered partnerships at later ages. Whether timing will account entirely for the most recent differentials can be established only when these younger cohorts reach their 40s and above.

**FIGURE 4:**
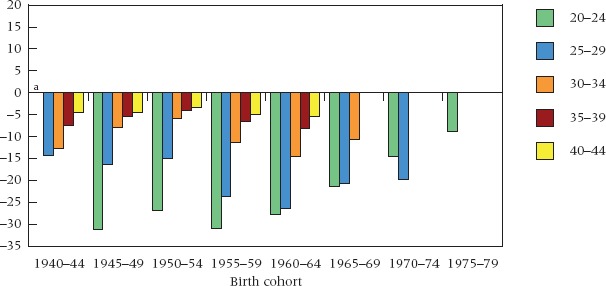
Educational differentials in ever marriage by birth cohort and age: Great Britain, cohorts 1940–44 to 1975–79 NOTE: See note to Figure 1. ^a^Data unavailable for ages 20–24 in 1940–44 birth cohort. SOURCE: Same as Table 1.

Timing differentials of this kind are also characteristic of female marriage in Britain (Figure 4). Among women born up to 1960–64, proportions ever married at ages 20–24 among the less educated exceeded those among the best educated by at least 25 percentage points. By age 40–44, however, cumulative proportions ever married among the best educated were almost as high as among the less educated, and the gap was no larger than 5 percentage points in any cohort. The decline in the gap with rising age is a classic reflection of a timing difference. It is not new. More and earlier marriage among women either less educated or of lower social status backgrounds has been a longstanding feature of Western societies for at least a century (Hajnal 1954; Tietze and Lauriat 1955; Grebenik and Rowntree 1963; Isen and Stevenson 2011). In this context, it seems reasonable to expect that the broadly similar age pattern of cohabitation differentials emerging in the most recent cohorts (Figure 3) will ultimately reflect mainly a timing difference also. In other words, following a period of innovation and diffusion, the social differential in timing traditionally characterizing female marriage is now being reinstated in relation to cohabitation.

**FIGURE 5:**
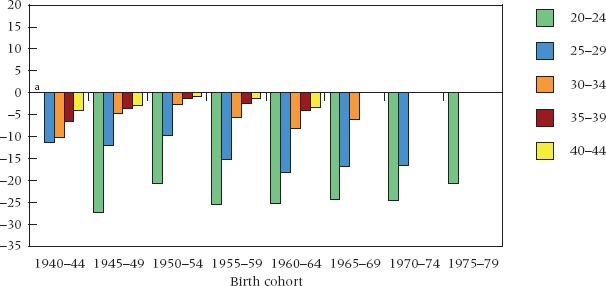
Educational differentials in cumulative incidence of partnership (cohabitation or marriage) by birth cohort and age: Great Britain, cohorts 1940–44 to 1975–79 NOTE: See note to Figure 1. ^a^Data unavailable for ages 20–24 in 1940–44 birth cohort. SOURCE: Same as Table 1.

We saw a pronounced switch over time in cohabitation differentials in Figure 3 and a diminution in the differentials in the cumulative incidence of marriage in recent cohorts in Figure 4. But when the two types of union are combined, social differentials appear to have changed very little (Figure 5). Within each cohort, many more women in the low education group have been in a partnership at younger ages, but by the early 40s the best educated have caught up: once again, the classic timing effect. There is, however, little change in the age profile of differentials across cohorts in Figure 5, unlike what we saw in Figure 3 and unlike the narrowing of differentials in recent cohorts in Figure 4. In the case of partnerships as a whole, a remarkable stability in social patterns emerges. Despite rapid change, there is much underlying continuity.

Finally, we return to the cross-sectional proportions cohabiting among women currently in a union. We saw in Figure 1 that in 2000–04 this conditional probability was higher among the best educated at ages under 35. This has been true of women in their 20s since the early 1980s: among those in a union at any given time at these ages, more of the best educated were cohabiting than of the least educated.

## Summary and discussion

A number of studies have reported or cited an inverse association between education and cohabitation. In three respects this association does not hold in the British case. First, retrospective GHS partnership histories reveal that unmarried cohabitation began in the 1970s and 1980s among the best rather than the least educated women.^16^ In the cohorts of 1945–49 to 1955–59, women leaving education at later ages had the highest cumulative incidence of cohabitation in virtually all age groups. Second, starting with the cohorts of the early 1960s, cumulative proportions ever cohabiting among the less educated began to approach those of the best educated; and in the most recent cohorts, the less educated have exceeded the best educated in the proportions ever having cohabited at young ages. Importantly, however, it appears that differentials by age in the latest cohorts will ultimately represent mainly a timing effect. That is, current trends suggest that the proportions ever cohabiting in each education group will be very similar by age 40–44, but with the less educated simply having started cohabiting earlier than the better educated. Third, for women in their 20s who, at any point in time, are in a union, the best educated are more likely to be cohabiting than the less educated.

We saw also that timing is a prominent feature of educational differentials in ever marriage and that this is of very long standing.^17^ The phenomenon must be attributable at least in part to the later age at which the best educated leave education. And although the highly educated were early adopters of unmarried cohabitation in Britain, the underlying determinants of social marriage patterns seem to have been reasserting themselves and restoring longstanding differentials in partnership formation. In times past, as now, women with low education married earlier than those with higher education. Now, as then, the less educated enter partnerships of all kinds—cohabitation and marriage—earlier than the better educated. This traditional pattern is often forgotten in commentary on social differentials in cohabitation; the same is true of nonmarital childbearing (England, Shafer, and Wu 2012). There is a great deal more continuity with the past than may be apparent when the focus is exclusively on cohabitation and when data are limited to a single cohort or time period (Santow and Bracher 1994; Bracher and Santow 1998).

## Comparative evidence

How far the three key findings of this article—early adoption of cohabitation by better educated women in Britain, the subsequent reversal in educational differentials in proportions cohabiting, and the current appearance of mainly a timing difference in cohabitation between education groups—apply in other developed countries needs further investigation. Evidence on reversal is patchy. One Swedish study concluded that modern cohabitation originated among both the working class and the social elite (Blom 1994). Several European sources suggest the initial differential in the 1970s whereby women who were either well educated or from advantaged backgrounds had higher proportions cohabiting diminished or reversed in later years (Roussel and Bourguignon 1978; Villeneuve-Gokalp 1991; Manting 1996; Ermisch and Francesconi 2000; Prioux 2009). A handful of American studies suggest that cohabitation was more common among the best educated in the US in the 1970s and early 1980s (Glick and Spanier 1980; Bachrach 1987; Goldscheider and Goldscheider 1999: 158–159). But, because more recent US sources (cited above) generally report an inverse association between education and cohabitation, this raises the possibility that a reversal of differentials of the kind documented here for Britain may also have occurred in the US (see also Sassler and Goldscheider 2004). However, American data on cohabitation in the early 1980s and before are of uncertain quality (Casper and Cohen 2000; Fitch, Goeken, and Ruggles 2005; Hayford and Morgan 2008).

Comparable evidence on the role that timing may play in group differences in education is not yet available for other countries. The reasons for this gap in the literature are largely methodological. Descriptive data on education differentials in cohabitation are often presented either for a broad age group such as 19–44 or for a single age group such as 25–29 (see, e.g., Kennedy and Bumpass 2008; Esteve, Lesthaeghe, and López-Gay 2012). A timing difference between groups cannot be identified with such data. To see a timing effect, differentials in the cumulative incidence of cohabitation at successive ages need to be examined; it will be most readily apparent in cohort format. In multivariate studies of entry into cohabitation, an interaction term between education and age would be required to detect a timing effect, but this is rarely employed.^18^ Nevertheless, one of the most systematic findings of recent multivariate investigations across contemporary developed societies is the sharp reduction in union formation rates associated with educational enrollment. As a result, it seems likely that timing differences also play a greater or lesser part in educational differentials in union formation in many other countries.

## Two additional questions

Two questions, both substantive and methodological, are raised by our findings on the changing relationship between education and cohabitation. The first is why it was the better educated in Britain who pioneered modern cohabitation. The explanation often offered is that the well educated were in the vanguard of value change, embracing nonconformist and anti-authoritarian attitudes and rejecting traditional marriage as outmoded—in short, an explanation rooted in the cultural change associated with the theory of the second demographic transition (Lesthaeghe 1995; Manting 1996; Surkyn and Lesthaeghe 2004).

Another explanation for the early adoption of cohabitation by the better educated is that better-educated women had more opportunity to enter unmarried cohabitation for two main reasons. First, as we saw above, well-educated women have traditionally married at older ages than the less educated. As a result, they will have spent more time single in early adulthood. Thus, when the barriers to unmarried cohabitation began weakening in the 1960s and 1970s, proportionately more of the well educated than of the less educated were single in young adulthood. They were therefore freer to cohabit than their less educated counterparts who, having married at young ages, were not in a position to cohabit. In analyses not reported here we found that in the early part of the period the propensity of well-educated women to enter cohabitation exceeded that of the less educated, so our results are not purely attributable to the higher proportions unmarried among the well educated. A second potential contributory factor is that, given the nature of the British higher education system, more of the well educated will, as students, have lived away from their parents at young ages. They will thus have been freer of parental supervision and community norms in young adulthood.^19^ Finally, the best educated may have had more access to efficient contraception in the form of the pill, and thus have been more secure in their ability to avoid pregnancy when cohabiting.

A second substantive question is to explain why, throughout the period examined, among those in a union in their 20s the better educated were more likely to be cohabiting than were the less educated (for similar findings see, e.g., de Jong Gierveld and Liefbroer 1995; Prioux 2009). Timing may be part of the explanation. Because the best educated complete their education and training at a later age, they are, at any given time in their 20s, at a younger social age than people who left school early (Skirbekk, Kohler, and Prskawetz 2004; Ní Bhrolcháin and Beaujouan 2012). They are thus less advanced in their partnership experience than early school leavers, and so less likely to have made the transition from cohabitation to marriage. Throughout the period considered, among those who had ever been in a partnership, proportionately more of the early leavers than late leavers had been married. This is the case both because of an earlier timetable and because the less educated have more often married directly, without first cohabiting (see Ní Bhrolcháin and Beaujouan 2013: Figure 8c).

## Interpretation and methods

In Britain in the most recent period considered, the large majority of women in all education groups in their early 30s had cohabited at some stage, and the upward trend was continuing. Periods of cohabitation are fairly short, with just one in ten lasting a decade or more (Beaujouan and Ní Bhrolcháin 2011). Most cohabiters—four in five in the UK—still eventually marry either their cohabiting partner or another partner. Two observations follow from this. First, a binary classification of women as cohabiters and non-cohabiters is inaccurate and potentially misleading: cohabitation is not, for most, a lifetime alternative to marriage. Second, it is inaccurate to label cohabitation the “poor man’s marriage,” as is sometimes suggested (Oppenheimer 2003; Smock and Manning 2004; Kalmijn 2011). If the term marriage is to be used, it would be more accurate to describe cohabitation as a young man’s marriage, and a young woman’s too, or alternatively the union of people with uncertain economic prospects, regardless of educational level (Landale and Forste 1991). In the GHS, people who were cohabiting at the time of the survey were on average ten years younger than those who were married—a sizable gap, and an unsurprising one, given the predominant role of cohabitation as an early stage in the life course.^20^

Finally, where there is a substantial timing element, we question the appropriateness of referring to a negative educational gradient in cohabitation in younger age groups without drawing attention to its role as a component of a timing effect. We suggest also that any inverse association between education and cohabitation should, for completeness, be put in the context of the negative educational gradient in marriage at younger ages.

## Concluding comments

Cohabitation is a dynamic process, both in the individual life course and over time. The social acceptability, frequency, place in the life course, and social patterning of cohabitation have been changing over time (Manting 1996; Smock 2000; Raley 2001; Seltzer 2004). A full understanding of its historical, demographic, and policy significance may not be possible until the transformation in the status of cohabitation from innovation to a normal part of the life course is complete. That transformation is still underway in Britain. In all probability, the speed of change and the stage reached in this historical process vary among countries. For example, cross-national differences in the link between cohabitation and individual attributes such as education may be due in part to countries being at different stages of a historical change similar to that seen in Britain. Links between partnership behavior and social and economic characteristics are often interpreted as though static. Our findings show that such stability cannot be assumed.

Following a transitional period of innovation and diffusion, contemporary cohabitation and partnership are reproducing social patterns traditionally exhibited by marriage. Any explanation of more frequent and earlier partnership among less educated women needs to account for historical as well as contemporary patterns.

## Footnotes

1 Common law marriage is a precursor phenomenon but appears from historical censuses not to have been widespread in the United States in the nineteenth century (Casper and Cohen 2000; Fitch et al. 2005). British historical evidence is patchy and subject to debate (Thane 2010; Probert 2012).

2 Barlow (2004); Liefbroer and Dourleijn (2006); Law Commission (2007); Goodman and Greaves (2010); Crawford et al. (2012); Perelli-Harris and Gassen (2012).

3 Durham (2001); Skolnick (2004); Barlow et al. (2008); Cook (2010); Hayton (2010); Taylor, Morin, and Wang (2011); Haskin, Coontz, and Fasman (2012).

4 Bumpass and Sweet (1989); Rindfuss and VandenHeuvel (1990); Thornton, Axinn, and Teachman(1995); Bumpass and Lu (2000); Smock and Manning (2004); Kennedy and Bumpass (2008); Koytcheva and Philipov (2008); Cherlin (2010). Clayton and Voss (1977) suggested that cohabitation was more frequent among the less educated as early as the 1970s in the US, but their study appears to confound education and race.

5 Roussel and Bourguignon (1978); Glick and Spanier (1980); Spanier (1983); Bachrach (1987); de Jong Gierveld and Liefbroer (1995); Kiernan and Lelièvre (1995); Kiernan (2004).

6 Lillard, Brien, and Waite (1995); Clarkberg (1999); Sassler and Goldscheider (2004); Hoem (1986); Lesthaeghe and Moors (1994); Santow and Bracher (1994); Leridon and Toulemon (1995); Manting (1996); Bracher and Santow (1998); Berrington and Diamond (2000); Billari et al. (2002); Nazio and Blossfeld (2003); Francesconi and Golsch (2005); Kalmijn and Luijkx (2005); Mills (2005); Hango and Le Bourdais (2007); Bradatan and Kulcsar (2008); Gabrielli and Hoem (2010); Gerber and Berman (2010); Kalmijn (2011).

7 Villeneuve-Gokalp (1991); Manting (1996); Goldscheider and Goldscheider (1999, pp. 158–159); Ermisch and Francesconi (2000); Prioux (2009).

8 Smock (2000); Kiernan (2004).

9 Specifications include the transition to first cohabitation, with or without marriage as a competing risk, or the probability that a first union is a cohabitation.

10 Some investigations focus only on younger ages up to the early or mid-20s (e.g. Bumpass, Sweet, and Cherlin 1991; Thornton, Axinn, and Teachman 1995; Schoen et al. 2009) or to 31 (e.g. Clarkberg 1999; Xie et al. 2003); some focus on a single age group (e.g. Kiernan 2004; and Esteve, Lesthaeghe, and López-Gay 2012 examine those aged 25–29 only); and others report differentials for a single broad age range such as 19–44 (e.g. Bumpass and Lu 2000; Kennedy and Bumpass 2008; Smock, Casper, and Wyse 2008; Manning 2010).

11 A time-series database of annual General Household Survey (GHS) rounds from 1979 to 2007 is the basis for the present analysis. It incorporates harmonized histories of fertility, marriage, and partnership collected in each round of the GHS over that period. Full details are given in Beaujouan and Ní Bhrolcháin (2011). From 2000–01 onward, the GHS recorded the dates of the start and end of up to seven marriages, the start date of any premarital cohabitation preceding each marriage, the start and end date of up to three cohabitations that had not ended in marriage, and the start date of any partnership, whether marriage or cohabitation, current at interview. The marriage histories correspond closely to vital registration statistics. Cohabitation histories, too, are of good quality, giving retrospective estimates just slightly above those implied by cross-sectional GHS figures. Our estimates of the prevalence of cohabitation are consistent with indirect estimates based on vital registration sources, and somewhat below those given by the British Household Panel Survey.

12 The sample analyzed is confined to women with valid partnership and fertility histories and a valid age at leaving education. Of an initial sample of 58,155 women aged under 60, 8.2 percent were proxy respondents or refused to answer the Family Information section and were therefore omitted. A further 3.7 percent were omitted because of irrecoverable errors in the partnership or fertility histories, and 1.6 percent because of missing information on the age at completing education.

13 The age at completing continuous education reported in GHS rounds from 2000–01 onward corresponds closely to national figures on full-time education or training (Ní Bhrolcháin and Beaujouan 2012); for simplicity we refer throughout to the age at completing education. The GHS questions on education are not detailed enough to establish how people responded who took a gap year between leaving secondary education and starting college or university. However, the data suggest that those who did so are more likely to have reported the age at which they left college or university rather than secondary school. This is because the estimates of age-specific educational participation are somewhat above official estimates of full-time education throughout the period examined (see Ní Bhrolcháin and Beaujouan 2012).

14 An extended version of the present article (Ní Bhrolcháin and Beaujouan 2013) gives confidence intervals around the estimates presented in both text and figures.

15 It may seem that the positive educational differential is anomalous, given the general supposition that, in the past, common law marriage was essentially a working-class phenomenon. However, estimates of the prevalence of common law marriage in the 1960s and before put it at between 0.5 percent and 2.2 percent of couple households in the US (Fitch, Goeken, and Ruggles 2005). British sources are less robust but also suggest rarity (see note 1). Because such unions were infrequent, it seems likely that they would have been of minor importance relative to the rise in cohabitation that began during the 1960s.

16 For related evidence from the British Household Panel Survey, see Ermisch and Francesconi (2000).

17 The less educated have a higher cumulative incidence of ever marriage at younger ages. Although the gap has been diminishing in recent cohorts, it has neither closed nor crossed over. These figures do not support the concern expressed in recent years in conservative policy circles that marriage is becoming the preserve of the middle class in Britain (Watt and Wintour 2009; Wintour and Watt 2009; Centre for Social Justice 2012). In the US, although it has been forecast that college-educated women would soon exceed the less educated in proportions ever marrying (Goldstein and Kenney 2001), that reversal of historical patterns has not yet occurred. Fewer white college-educated than non-college women currently in their 50s in the US have married, although the gap appears to have closed among white women in their mid-30s. Among black women, however, the college educated are more likely ultimately to marry (Fry 2010; Isen and Stevenson 2011).

18 Forthcoming analyses for several developed countries by Karel Neels and Brienna Perelli-Harris provide evidence confirming our results both on the reversal of educational differentials in cohabitation across cohorts and the existence of a timing effect.

19 A UK study (Berrington and Diamond 2000) and two studies of the Netherlands (Liefbroer 1991; Manting 1996) found that young people living at home were much less likely to enter cohabitation than were those living elsewhere. While this is also true of marriage, the effect appears to be larger for cohabitation in earlier cohorts.

20 The ten-year difference relates separately to men and to women. It is based on a sample of men and women aged 16–59 who answered the Family Information section of the GHS in 1986–2007. The gap is close to constant over that period for each sex. In the adult population as a whole, the difference would be larger.
